# Supplementary material for: Community Stakeholders’ Perspectives on Intimate Partner Violence during Pregnancy—A Qualitative Study from Ethiopia
Source: Int J Environ Res Public Health. 2019 Nov 25;16(23):4694. doi: 10.3390/ijerph16234694 (PMC6926756; doi:10.3390/ijerph16234694)
Supplement: Supplementary file 1 [file ijerph-16-04694-s001.pdf]

Table S1. Procedures/steps of data analysis in Atlas Ti, based on the content analysis, and framed by the theory of Gender and Power

Steps	procedures and descriptions
Familiarizations	All transcripts were read to make a general sense out of it and reflect on the overall meaning
Creating and file naming	Create file name and save the project (under new hermeneutic unit/ analysis project) and add document in the library
Importing files	Import all transcripts (#16 IDIs transcripts lined up under P-Docs)
Condensed meaning unites	Open each transcripts turn by turn and create meaning unites that gives sense
Coding	Create codes/label (under code manager)<br>Highlight quotations for each transcripts, then drug and drop codes for each respective quotations, or create new codes as necessary
Categorizing	Categorize/create family codes (themes) based on similarity<br>Write comments under codes and memos under family as appropriate
Concept mapping	Semantic link/networking codes to codes help us to have a concept maps, and quotation to quotations to have an argument maps
Producing outputs (categories /families)<br>Data familiarization	Open code manager, click on each codes, click on outputs and save each outputs of the codes of categories /families<br>For more data familiarization, transcripts were read repeatedly, alongside listening to records of recordings and /or reading field-notes
Result (describing themes and quotations	In the result section-a thorough description of each output of categories under the respective themes, based on the content analysis method selected for this specific analysis. Supporting quotations were included within the respective themes.
Discussion	In the discussion-interpret results; discuss them in light of relevant literatures within the field of IPV as well as Connell's theory of gender and power.
